# Editorial: Risk Stratification Strategies for Cardiac Rhythm Abnormalities

**DOI:** 10.3389/fcvm.2022.887461

**Published:** 2022-04-27

**Authors:** Gary Tse, Nan Zhang, Wenhua Song, Konstantinos P. Letsas, Tachapong Ngarmukos, Kamalan Jeevaratnam, Tong Liu

**Affiliations:** ^1^Cardiac Electrophysiology Unit, Cardiovascular Analytics Group, Hong Kong, China; ^2^Tianjin Key Laboratory of Ionic-Molecular Function of Cardiovascular Disease, Department of Cardiology, Tianjin Institute of Cardiology, Second Hospital of Tianjin Medical University, Tianjin, China; ^3^Kent and Medway Medical School, Canterbury, United Kingdom; ^4^Arrhythmia Unit, Onassis Cardiac Surgery Centre, Athens, Greece; ^5^Faculty of Medicine Ramathibodi Hospital, Mahidol University, Bangkok, Thailand; ^6^Faculty of Health and Medical Sciences, University of Surrey, Guildford, United Kingdom

**Keywords:** atrial fibrillation, risk stratification, cardiac rhythm abnormalities, biomarkers, prediction model

## Introduction

Cardiac rhythm abnormalities, such as atrial fibrillation (AF) and ventricular tachycardia (VT)/ventricular fibrillation (VF), account for a significant proportion of adverse cardiac events and mortality (1). In fact, most cardiovascular pathologies will lead to some form of rhythm abnormalities as the pathology worsens. In many instances such rhythm abnormalities precede the fatal event. Accurate risk stratification is central to early and timely treatment in high-risk patients and to avoid unnecessary invasive procedures. There are protocols and diagnostic algorithms currently in place that help clinicians to profile the risk associated with a condition. None of these tools is fully accurate despite ongoing efforts to improve risk stratification strategies (2). Current clinical practice involves a combination of clinical history, genetic testing, non-invasive electrocardiographic measurements (3) and invasive electrophysiological studies (4). The aim of this Research Topic is to (1) investigate the physiological mechanisms that underlie cardiac rhythm abnormalities, (2) examine current approaches used for risk stratification in different conditions and their impact on patient outcomes, and (3) explore the use of experimental models and computational and machine learning algorithms to facilitate the development of risk stratification tools.

## Pre-Clinical Models for Studying Cardiac Arrhythmias

Cellular, animal and computational models have been used to study the molecular and electrophysiological mechanisms underlying cardiac arrhythmias (5). Saadeh and Fazmin review the mechanisms by which age-related mitochondrial dysfunction promotes arrhythmic triggers and substrate. This provided insights into novel potential anti-arrhythmic pharmacological interventions that specifically target upstream mitochondrial function, and hence ameliorates the need for therapies targeting downstream changes which have constituted traditional antiarrhythmic therapy. Deng et al. reviews the key role of uric acid in mediating oxidative stress and inflammation, which underlie fibrotic change of the atria predisposing to AF. Fong et al. conducted a meta-analysis into modulated calcium homeostasis and calcium release events in AF, demonstrating higher sarco/endoplasmic reticulum Ca^2+^-ATPase (SERCA) expression in the between primary diseased AF group, but lower expression in the secondary AF groups. Costa et al. describe in-depth the molecular interactions between sex hormones and the cardiac ion channels, as well as the clinical implications of these interactions on the cardiac conduction system, in order to understand the link between these hormones and the susceptibility to cardiac arrhythmias.

Avula et al. presented APD dispersion data from two-dimensional optical mapping in a mouse model with F1759A SCN5A overexpression modeling long QT syndrome type 3. They then presented theoretical models for APD dispersion, methods for analysis and calculation of APD dispersion, and showed that APD dispersion in clustered patterns is the predominant configuration for spontaneous occurrence and persistence of AF and VF. Liu et al. conducted experiments to examine the effects of autonomic input in vein of Marshall-mediated AF. They found that electrical stimulation of the left superior ganglionated plexi shortened atrial refractoriness, increased APD dispersion and the vulnerability window. These effects were prevented by low level stimulation or ethanol ablation of the vein of Marshall, or muscarinic blockade by atropine. Acquired causes of VT/VF may arise from remodeling after myocardial infarction. In a mouse model with knockout of the CC chemokine receptor 9 (CCR9), abnormalities in ion currents, calcium handling, gap junction and action potential conduction were prevented, suggesting that the CCR9 can be a promising therapeutic target for reducing the likelihood of myocardial infarction-related arrhythmias (Huang Y. et al.).

## Risk Stratification for Cardiac Arrhythmias

AF is the commonest cardiac rhythm abnormality observed in clinical practice and accounts for significant morbidity and mortality *via* the development of ischemic stroke (6), dementia (7, 8) and heart failure (9). Shang et al. reviewed the clinical applications and utility of AF characteristics, cardiac imaging and electrocardiogram markers, arterial stiffness and atherosclerosis-related markers, circulating biomarkers, and novel genetic markers for the diagnosis of ischaemic stroke and non-valvular AF. Ning et al. reviewed the current literature on the etiology of atrial cardiomyopathy in embolic strokes of undetermined source. Liao L.-Z. et al. conducted a two-sample Mendelian randomization study demonstrating a causal inference between hypertension and AF. Hidru et al. conducted a cohort study of 9,618 hypertensive patients and identified serum uric acid levels and left atrial diameter as significant predictors of AF. Moreover, the modified Taiwan AF score consisting of age, male gender, hypertension, heart failure, coronary artery disease and end-stage renal disease predicted incident AF in a Chinese population with an area under the curve of 0.86 for 1-year follow-up, 0.83 for 5-year follow-up, 0.80 for 10-year follow-up, and 0.75 for 16-year follow-up (Liao J.-N. et al.).

One of the most devastating outcomes of AF is stroke for which the CHA2DS2-VASc score has been used for its risk stratification (10). A recent study explored low risk patients with CHA2DS2-VASc scores of 0 to 1 (Kim, Yu, Kim, Lee et al.), demonstrating that in patients with normal left ventricular ejection fraction, high H2FPEF score and increasing age were independently associated with the development of ischemic stroke. Song et al. found that a comprehensive evaluation of serum uric acid and B-type natriuretic peptide levels, left atrial diameter and left ventricular ejection fraction can stratify the risk of stroke in patients with non-valvular AF. A large retrospective cohort study conducted propensity score matching between with atrial flutter and AF (Wang H.-T. et al.). Among patients without history of stroke, the risk of dementia was higher in patients with AF than in patients with AFL.

The following studies investigated the impact of interventional and medical treatment on outcomes in patients with AF. Balloon-based catheter ablations, including hot balloon ablation and cryoballoon ablation, have rapidly emerged as alternative modalities to conventional catheter ablation owing to their procedural advantages and better clinical outcomes and safety profiles. A recent study found that patients undergoing hot balloon ablation had a higher incidence of touch-up ablation and longer procedural time, but with comparable clinical outcomes on mid-term follow-up (Peng et al.). patients with short episodes of AF <24 h were compared to those with 24 h or longer in a propensity score-matched analysis for cryoablation of pulmonary veins isolation (Jiang et al.). Higher success rate and lower incidence of stroke/transient ischaemic attacks were observed for the <24 h group compared to the ≥24 h group during follow-up. Recurrence in AF remains a problem despite improvement in ablation technology. A prospective cohort study found that leukocyte telomere length predicted disease progression from paroxysmal to persistent AF following catheter ablation (Wang Q. et al.). Soluble Suppression of Tumorigenicity 2, was shown to be predictive of AF recurrence after radiofrequency ablation in patients with persistent AF (Tan et al.). A systematic review and meta-analysis found that the HAS-BLED score has moderate predictive abilities for bleeding risks in patients with AF regardless of type of oral anticoagulants (Gao et al.). For medical treatment of AF, beta blockers are frequently used. Interestingly, beta blockers consistently decreased long-term mortality in patients with high-burden and low-burden of premature atrial complexes, and this effect appears not to be mediated through a reduction in AF or new onset stroke (Huang T.-C. et al.). Recent work has explored traditional Chinese medicine as an adjunct therapy (11, 12). A systematic review and meta-analysis of randomized controlled trials on Zhigancao Decoction combined with metoprolol was performed, demonstrating good efficacy with few adverse events for different types of arrhythmias that include AF (Yang et al.).

A registry study from Shanghai, China investigated the impact of new onset AF in patients with acute myocardial infarction. Interestingly, the authors found that asymptomatic rather than symptomatic new onset AF was significantly predictive of cardiovascular mortality and all-cause mortality (Luo et al.). In a propensity score-matched study between patients undergoing transcatheter and surgical aortic valve replacement, post-operative AF had a worse impact on heart failure-related hospital admissions and composite outcome of mortality, stroke and heart failure-related admissions (Jeong et al.). In a single center prospective study, machine learning models were developed using least absolute shrinkage and selection operator (LASSO) and random forest (RF) algorithms for variable selection (Chen Y. et al.). Their LASSO-Cox model showed an area under the curve of 0.84 for predicting 1-year mortality.

Age was identified as a risk factor of syncope recurrence in elderly patients with vasovagal syncope and positive head-up tilt test (Guo et al.). Another study from the same group examined a large cohort of 4,873 patients undergoing the head-up tilt test, finding that direct drug potentiation is safe and sensitive for diagnosing vasovagal syncope (Xu et al.). Patients with an overlap syndrome between chronic obstructive pulmonary disorder and obstructive sleep apnoea showed higher rates of cardiac arrhythmias (such as AF, premature atrial contraction, ventricular premature contraction, and atrioventricular or VT) compared to those with either condition alone (Tang et al.). Heart rate was shown to be predictive of all-cause mortality in patients suffering from multiple myeloma, but further work is needed to explore the prognostic role of cardiac arrhythmias in this condition (Wang J. et al.). Li and Zhang developed an autodetection algorithm using neural network combined with the attention-based bidirectional long-short term memory model for classifying nine different types of ECG patterns, including normal electrocardiogram, atrial fibrillation, atrioventricular block, left bundle branch block, right bundle branch block, premature atrial complexes, premature ventricular complexes, ST depression and ST elevation (Li and Zhang). Regarding AF treatment, the H2FPEF Score, which reflects the degree of left ventricular diastolic dysfunction, was improved following catheter ablation (Kim, Yu, Kim, Uhm et al.). Zhang et al. compared the catheter ablation to pacing in patients with tachycardia-bradycardia syndrome, reporting lower rates of the composite endpoint of cardiovascular-related hospitalization and thromboembolic events, as well as the progression of atrial fibrillation and heart failure.

VT/VF can arise from acquired causes, such as ischaemic heart disease, electrolyte disturbances and drugs. Tse, Li et al. reviewed the electrophysiological mechanisms that predispose to the development of atrial and ventricular arrhythmias by non-reentrant and reentrant mechanisms in hypokalaemia, a common electrolyte abnormality in hospitalized patients. Moreover, Zhou, Zhao et al. identified a non-linear relationship between body mass index and VT/VF in Chinese patients with implantable cardioverter-defibrillators (ICDs). Another study from the same group investigated whether the obesity paradox in all-cause mortality is present among the Chinese population with an ICD but did not demonstrate its presence (Zhou, Sun et al.). Sun et al. found that compared to night-time heart rate of ≤ 50 or ≥70 bpm, heart rate between 50 and 70 bpm was associated with lower risks of ventricular tachyarrhythmias, appropriate ICD shocks, inappropriate ICD shocks, and all-cause mortality. A retrospective study from Hong Kong, China investigated Chinese patients who were hospitalized for acute heart failure, demonstrating that fragmented QRS was a significant predictor of VT/VF, sudden cardiac death and cardiovascular mortality (Chan et al.).

Alternatively, VT/VF can be due to inherited heart diseases such as cardiomyopathies (13) or ion channelopathies (14). Patients diagnosed with arrhythmogenic and dilated cardiomyopathy can harbor mutations in the phospholamban gene. In patients with phospholamban p.Arg14del mutational carriers, high procollagen type I carboxy-terminal propeptide to C-terminal telopeptide collagen type I ratios correlated with end-diastolic and end-systolic volumes, T-wave inversion and the presence of premature ventricular contractions (van der Voorn et al.). The commonest ion channelopathy globally is long QT syndrome, which is more common in Western compared to Asian populations (15). Nevertheless, Tse, Lee, Zhou et al. conducted a territory-wide study into the epidemiology of LQTS in a city of China, and found that the application of random survival forest technique significantly improved risk prediction for VT/VF compared to Cox regression.

Brugada syndrome (BrS) is characterized by coved or saddle-shaped ST segment elevation in the right precordial leads (16). It has a higher prevalence in Asia compared to western countries. However, risk stratification is difficult, especially in asymptomatic subjects (17). Both depolarization and repolarization abnormalities are hypothesized to underlie arrhythmogenesis in BrS. ECG indices that are manually measured have been explored for their ability to predict future arrhythmic events (18–20). Recent efforts have focused on the use of automated measurements to facilitate risk prediction in BrS (21). Thus, in a cohort of Chinese BrS patients, Tse, Lee, Li et al. identified ST slope as a novel predictor of ventricular arrhythmogenesis. The use of invasive programmed ventricular stimulation (PVS) has aided risk stratification although its sensitivity, specificity, positive predictive value and negative predictive value can differ depending on the protocols employed (22). Recent work has demonstrated that right ventricular outflow tract electro-anatomical abnormalities can predict VF inducibility (23).

Nevertheless, recent available evidence indicates that risk prediction is more accurate when a multi-parametric approach rather than rely on a single investigative method (24). Monasky et al. evaluated the role of genetic testing for risk stratification in BrS. They recommend that whole exome or whole genome testing and family segregation analysis should always be performed. Chen X. et al. studied the clinical and genetic characteristics of 104 probands with early repolarization syndrome, reporting its association with loss-of-function genetic defects in genes encoding the cardiac calcium channel. They identified a unique clinical entity characterized by decreased heart rate and QTc, as well as increased transmural dispersion of repolarization. In the case of the CACNA1C-P817S variant, impaired trafficking of the channel to the membrane contributes to the loss-of-function in the calcium channel.

Finally, several studies examined risk stratification strategies for other tachycardias and also bradycardias. For example, the role of baseline-corrected QT interval dispersion (QTcd) in predicting the effectiveness of metoprolol in pediatric postural tachycardia syndrome was examined (Wang Y. et al.). The pre-treatment baseline QTcd were significantly longer in responders treated with metoprolol compared to non-responders and that it was negatively correlated with SS after metoprolol treatment. The prenatal management for immune-associated congenital heart block in fetuses was reviewed, in particular issues pertaining to clinical management, including the roles of autoantibodies in its pathophysiology, diagnosis and prognosis (Liao H. et al.). A study of multiple myeloma patients from Xi'an, China found that approximately half of the patients suffered from cardiac arrhythmias (Li et al.). In particular, those with sinus bradycardia had lower incidences of all-cause mortality compared to those without it.

## Concluding Remarks

The articles collected under this Research Topic advance our understanding of risk stratification strategies for cardiac rhythm abnormalities, presenting recent progress on the pre-clinical, clinical and epidemiological studies on the different cardiac arrhythmias. There is a growing body of evidence supporting a more integrative approach by combining new and established computational and experimental/clinical approaches to improve our understanding and treatment of cardiac arrhythmias. With better artificial intelligence and advances in wearable technologies, the application of machine learning algorithms on large physiological and clinical datasets can advance this exciting research area. Together, these methods will no doubt be an important part of global efforts to tackle cardiac rhythm abnormalities.

## Author Contributions

GT, NZ, and WS: initial draft and critical revision of manuscript. KL, TN, KJ, and TL: critical revision of manuscript. All authors contributed to the article and approved the submitted version.

## Funding

The work was funded by grants from National Natural Science Foundation of China (81970270 and 82170327 to TL) and Tianjin Key Medical Discipline (Specialty) Construction Project.

## Conflict of Interest

The authors declare that the research was conducted in the absence of any commercial or financial relationships that could be construed as a potential conflict of interest.

## Publisher's Note

All claims expressed in this article are solely those of the authors and do not necessarily represent those of their affiliated organizations, or those of the publisher, the editors and the reviewers. Any product that may be evaluated in this article, or claim that may be made by its manufacturer, is not guaranteed or endorsed by the publisher.
